# Intravascular Coronary Imaging Update: Advances, Clinical Applications, and Future Directions

**DOI:** 10.1007/s11886-025-02243-6

**Published:** 2025-07-11

**Authors:** Sergio Waxman, Umair Bajwa, Minh Tran

**Affiliations:** 1 The Division of Cardiovascular Medicine, Newark Beth Israel Medical Center, 201 Lyons Avenue, Newark, NJ 07112 USA; 2 Rutgers New Jersey Medical School, Newark, USA

**Keywords:** Intravascular imaging, Intracoronary imaging, Intravascular ultrasound, Near-infrared spectroscopy, Optical coherence tomography

## Abstract

**Purpose of the review:**

We sought to review the various intracoronary imaging modalities (intravascular ultrasound, optical coherence tomography, and near-infrared spectroscopy) and the latest evidence supporting their use in clinical practice.

**Recent findings:**

There is an increasing body of evidence that demonstrates that imaging-guided strategies are superior to angiography alone to improve outcomes of percutaneous coronary intervention (PCI). An intriguing and promising application is use of these devices to identify and treat high-risk or vulnerable plaques. The application of these modalities in special clinical scenarios is also reviewed.

**Summary:**

Intravascular imaging provides information beyond the angiogram that can be used to improve patient outcomes during PCI. The emerging evidence has been incorporated into the most recent practice Guideline recommendations. Future research is underway to establish the benefit of intravascular imaging for detection and treatment of vulnerable plaques.

## Introduction

Intravascular imaging has transformed the management of coronary artery disease (CAD) by providing detailed insights into vessel wall pathology and plaque morphology—capabilities that go far beyond the traditional luminal assessment offered by coronary angiography. This review will discuss the evolution of intravascular coronary imaging, current modalities with their strengths and weaknesses, compare key trials and studies supporting their use, discuss guideline recommendations from the American College of Cardiology (ACC)/American Heart Association (AHA) and European Society of Cardiology (ESC)/European Association for Cardio-thoracic Surgery (EACTS), and outline emerging technologies to optimize patient outcomes.

**Fig. 1:**
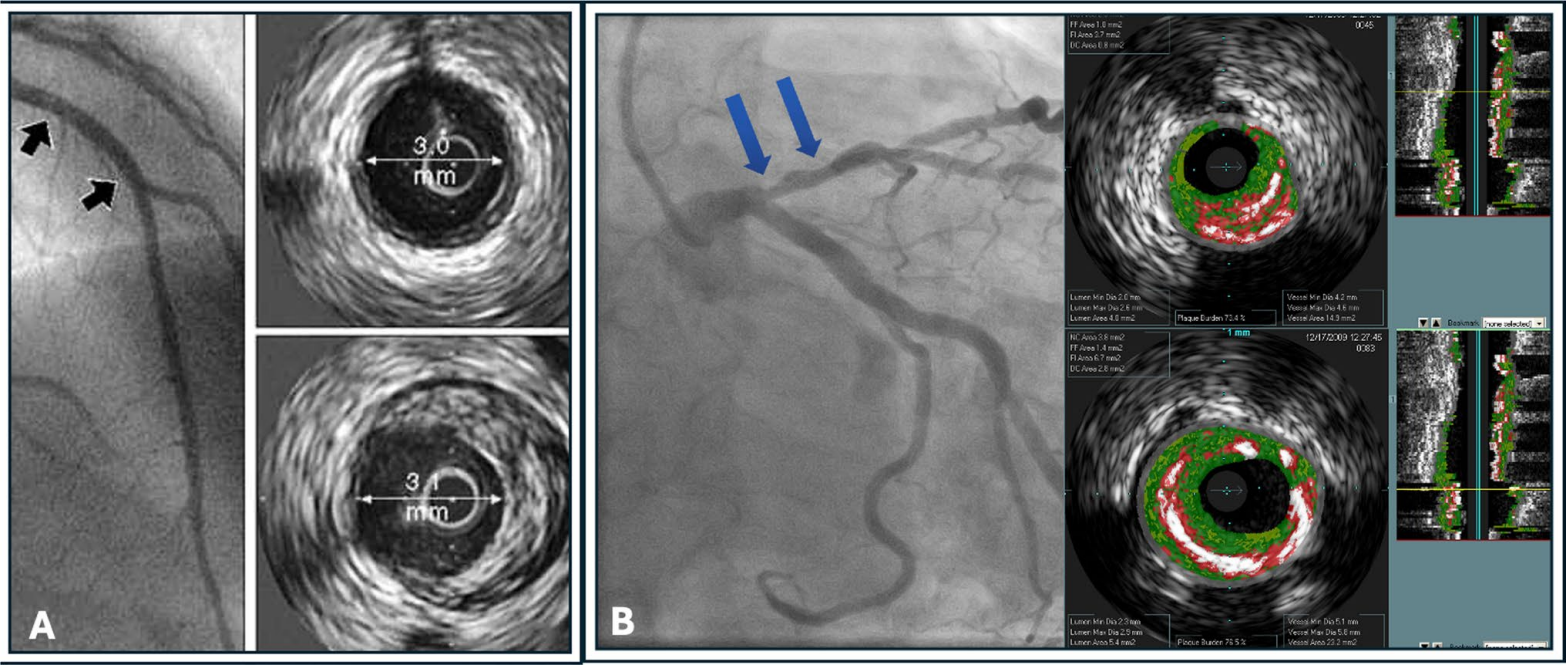
A. Representative coronary angiogram with corresponding IVUS image, demonstrating a) the limitation of angiographic lumenography to detect coronary atherosclerosis, especially in its earlier stages, and b) the process of remodeling described by Glagov, whereas there is outward growth of the vessel to accommodate plaque with preservation of the lumen (*black arrows*). (Panel A reproduced with permission from: Nissen S, Yock P. Circulation 2001;103(4):604–616, with permission from Wolters Kluwer Health, Inc.) [4]. **B**. Coronary angiogram with corresponding IVUS cross-sections (*blue arrows*), revealing an intermediate angiographic stenosis in the proximal left anterior descending artery. IVUS reveals plaque burden > 70%, MLA < 4mm^2^, and the presence of a VH-TCFA, all characteristics of a high-risk plaque as found in the PROSPECT Study [5]. Key: IVUS: Intravascular ultrasound. MLA: Minimal lumen area. VH-TCFA: Virtual histology thin-cap fibroatheroma

Since its serendipitous inception by Dr. Mason Sones in 1958, selective coronary angiography has guided the diagnosis and treatment of coronary artery disease [1]. Coronary angiography in living patients revealed snapshots of partially obstructed arteries, occluded vessels, and collaterals, which were correlated with the clinical presentations and syndromes of coronary atherosclerosis as we understand them today (asymptomatic CAD, stable and unstable angina, Non-ST elevation myocardial infarction (NSTEMI), and ST elevation myocardial infarction (STEMI). Meanwhile, histopathologic studies described the complexity and often-diffuse nature of the disease, challenging angiographic interpretations of lesion severity, focality, and even clinical significance. The description of vascular remodeling by Glagov in 1987 [2] and the recognition that luminal obstruction is a late and focal manifestation of a diffuse and chronic inflammatory process that occurs in the vessel wall [3], highlighted the limitations of lumino-angiography to detect and characterize atherosclerotic burden and lesions at risk of thrombosis (Fig. 1).

Yet despite its many limitations, angiography is still the mainstay for diagnosing coronary disease. Beyond the angiogram, advanced intravascular imaging modalities provide additional detail about disease morphology, vessel morphometry, and even function. The evolving promise of the intravascular imaging paradigm is that adding diagnostic information to the angiogram will translate into specific therapeutic interventions that affect clinical outcomes. The bulk of evidence is moving towards validation of this imaging paradigm and is the subject of this review.

## Brief Description of Approved Intravascular Coronary Imaging Modalities

**Fig. 2:**
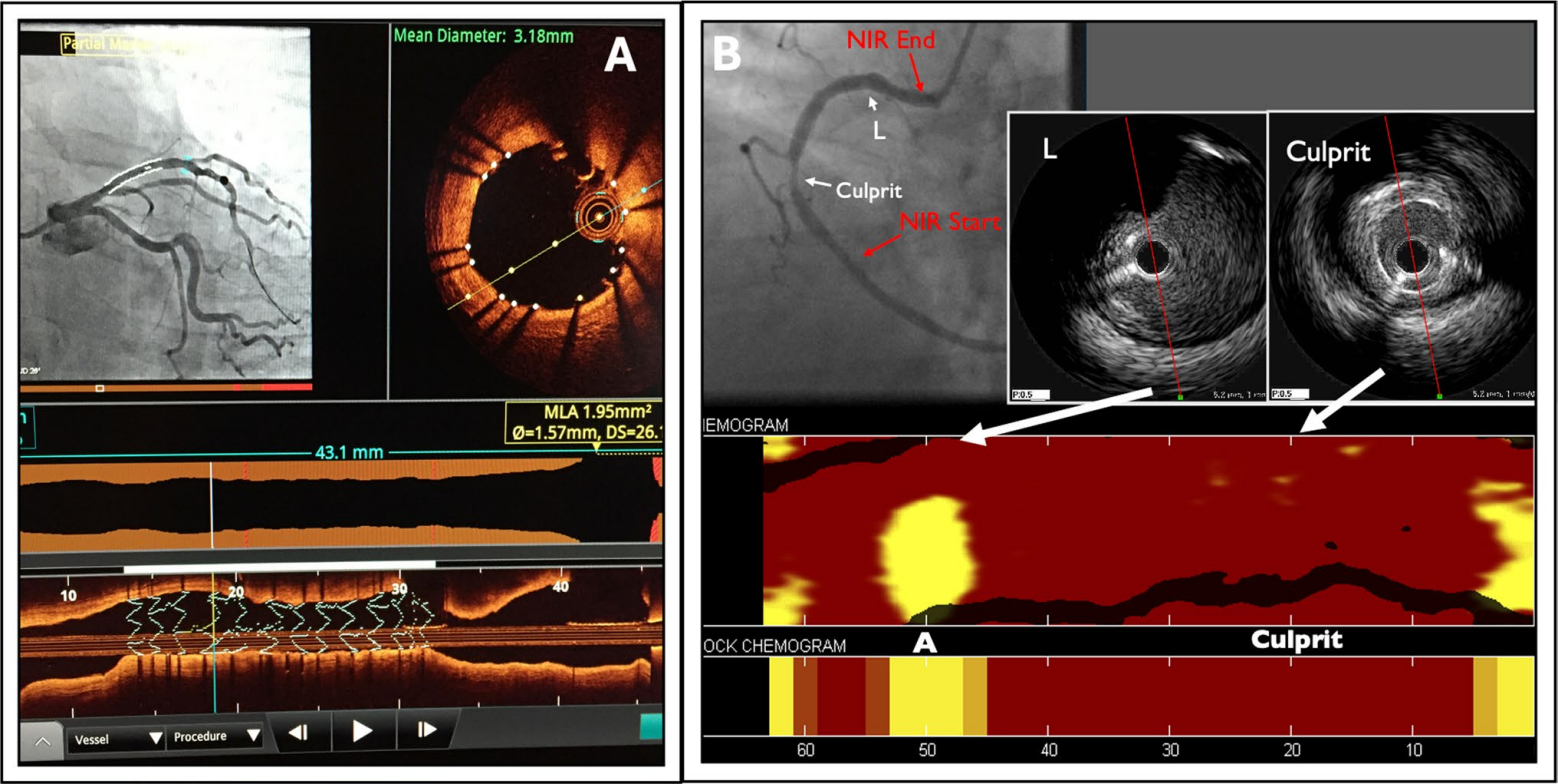
A. OCT display showing co-registration with the angiogram with its corresponding cross-sectional OCT frame at the point of the cursor (top). The stent struts (*white dots*) are well apposed to the vessel wall. Bottom part of the display shows longitudinal reconstruction of the artery with the stent in place and the luminogram that allows for precise measurements of lumen diameter, area, and lesion length. **B.** Right coronary angiogram still-frame with its corresponding chemogram obtained with NIRS-IVUS pullback. The culprit stenosis reveals significant lumen narrowing by IVUS but low probability of lipid by NIRS. A more proximal non-culprit area (L) reveals a high probability of lipid in a non-obstructive plaque by IVUS and angiography. Key: OCT: Optical coherence tomography. NIRS: Near-infrared spectroscopy. IVUS: Intravascular ultrasound

- Intravascular ultrasound (IVUS) has been the gold standard of intravascular imaging for over 3 decades. It uses ultrasound waves that are reflected differentially depending on the background tissue to generate a gray-scale, cross-sectional image of the vessel, and has axial and lateral resolution of 80–200 μm and 200–250 μm depending on the frequency used (20–40 MHz) [6]. It has a penetration of up to 8 mm and does not require blood clearance, which makes it ideal for assessment of plaque burden and vessel remodeling, as well as of ostial or very proximal lesions. Gray-scale IVUS is commonly used in clinical practice but is limited in assessing plaque composition. Virtual histology IVUS (VH-IVUS) enhances plaque characterization by analyzing backscattered radiofrequency signals, classifying tissue and creating a color-coded display that reflects dense calcium, necrotic core, fibro-fatty, and fibrous tissue [7] (Fig. 1). Because of its relative low resolution compared with optical coherence tomography, IVUS is less effective for visualizing the fine details of lesion pathology or of stent apposition and requires a high level of expertise for accurate interpretation.
- Optical coherence tomography (OCT) has emerged as an alternative to IVUS (Fig. 2). It uses a low-coherence light source and measures the intensity of light reflected from different depths within a tissue sample. These signals are then digitally converted into real-time high spatial and contrast resolution, cross-sectional, and 3-dimensional volumetric images, with near-microscopic axial and lateral resolution of 10 μm and 20 μm, respectively [6]. It has a penetration depth of 2 mm and requires blood displacement with a contrast/saline mixture. Its high resolution enables a more reliable assessment of lumen and vessel pathology, including thin-cap fibroatheroma, macrophages and neovessels, stent apposition and reendothelialization, thrombus, and dissection. Its relatively shallow depth of penetration and its reliance on blood clearance may preclude its use in ostial left main and right coronary artery lesions. New, higher penetration OCT systems are being developed that could eliminate the limitation of depth.
- Near-infrared spectroscopy (NIRS) is the only Food and Drug Administration (FDA)-approved modality for detection of lipid-rich plaque. It characterizes the chemical composition of tissue based on the differential absorption of light in the near-infrared spectrum. Captured spectral data are analyzed to produce a color-coded representation of the chemical composition of tissue indicating the probability of lipid in a longitudinal and quadrantic location within the vessel called a chemogram [8]. It is used in conjunction with either IVUS or OCT as an integrated dual-imaging catheter. Lipid-rich areas by NIRS have been associated with culprit lesions in STEMI, a higher risk of no-reflow during PCI, and future coronary events (Fig. 2).

**Fig. 3:**
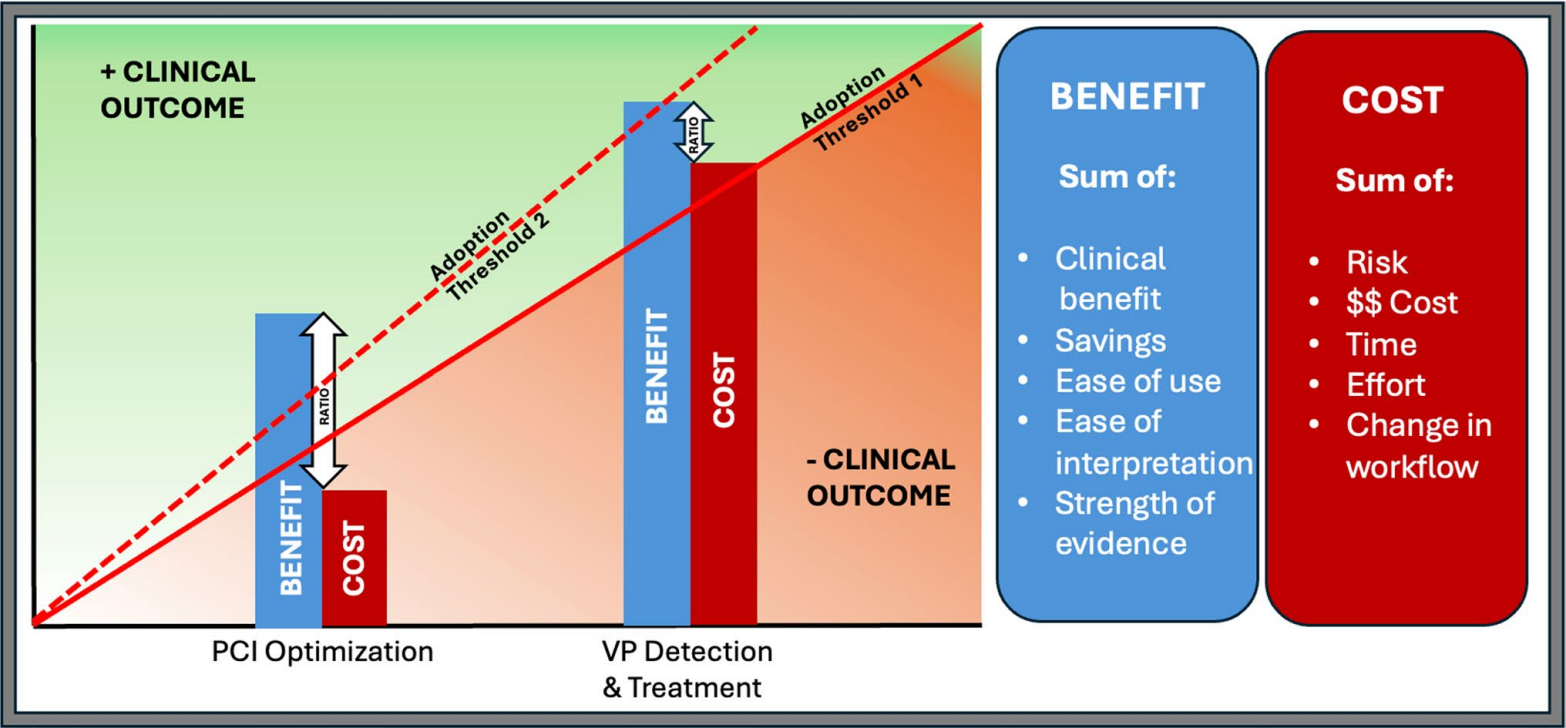
A theoretical framework for adoption of intravascular imaging for two different clinical applications. The sums of the benefits and costs are used to estimate the benefit/cost ratio of each application. The benefit/cost ratio of PCI Optimization is larger than that of VP Detection and Treatment despite a potentially lower clinical benefit (decrease in TLF vs potentially prevention of MI or death), since the risk of instrumentation of a culprit lesion, the incremental effort, and the current burden of proof of benefit are lower than addressing non-culprit disease even though the greater absolute benefit is potentially smaller. The threshold of adoption is dynamic and is affected by “cost” factors such as ease of use and interpretation, procedural time, and change to operators’ workflow related to imaging. The current lack of widespread adoption of intravascular imaging is likely related to this adoption threshold remaining high (*dotted line*) despite mounting clinical evidence. Further efforts need to be directed towards decreasing this threshold (*solid line*). Key: PCI: Percutaneous coronary intervention. VP: Vulnerable plaque. TLF: Target lesion failure. MI: Myocardial infarction

All three systems can now co-register intravascular imaging and angiographic data through nearly instantaneous postprocessing technology, producing a comprehensive coronary roadmap that can be used for clinical decision making and guidance of therapy. Co-registration can enhance procedural planning, reducing geographic miss during stent placement, and help optimize percutaneous coronary intervention (PCI) outcomes through comprehensive vessel assessment and precise stent deployment. Despite mounting evidence supporting use of imaging in distinct clinical scenarios, adoption remains relatively low, likely related to factors such as ease of use and interpretation that are being addressed to lower the threshold for utilization (Fig. 3). Table 1 summarizes key features of each modality, as well as key metrics associated with specific clinical applications.

**Table 1:** Comparison of IVUS, OCT, and NIRS

Variable	IVUS	OCT	NIRS	Selected References
Resolution	100–150 μm	10–20 μm	N/A	4
Imaging Depth	4–10 mm	1–3 mm	2–4 mm	
Requires Blood Displacement	No	Yes (mixture contrast/saline)	No	
Co-registration with Angio	Yes	Yes	No	
Key Advantages	Deep tissue penetration, real-time guidance	High resolution, detailed plaque characterization	Detects lipid core plaque, adjunct to IVUS or OCT	
Limitations	Resolution may hamper interpretation	Ostial disease, very large vessels	Limited indication as standalone modality	
Clinical Applications	PCI optimization, vulnerable plaque detection, assessment of lesion severity and ambiguous angiographic findings	PCI optimization, vulnerable plaque detection, assessment of lesion severity and ambiguous angiographic findings	Vulnerable plaque detection (lipid-rich plaque assessment)	
Parameters for PCI Optimization	MSLA ≥ 5.0 mm2 (proximal), ≥ 4.0 mm2 (distal); 90% of the MSLA at distal reference segment; Plaque burden < 50% within 5 mm of either stent edge; Stent expansion MSLA ≥ 5.5 mm2 or > 80% (MSLA/MRLA; No edge dissection > 60° arc, length > 2–3 mm, and flap extending to media/adventitia; MSLA > 7 mm2 for distal LM and > 8 mm2 for prox LM	MSLA > 4.5 mm2; No major stent malapposition (< 200 μm gap); No edge dissection > 60° arc, length > 2–3 mm, and flap extending to media/adventitia; MSLA ≥ 80%−90% of MRLA; MSLA > 7 mm2 for distal LM and > 8 mm2 for prox LM		4,10,17,18, 19,20,27,28
Parameters for Vulnerable Plaque Detection	Plaque burden ≥ 70%; MLA ≤ 4.0 mm2; VH-TCFA	Fibrous cap thickness < 65–75 μm; MLA < 3.5–4 mm2; Lipid arc > 180°- 270°; Presence of macrophages	Lipid Core Burden Index (Max LCBI in 4 mm segment) > 315–400	5,33,34,35,36
Parameters for Assessment of Functional Significance of Intermediate Lesions*	Vessel dependent (MLA < 3.0 mm2 in LAD, MLA < 2.4 mm2 in non-LAD); MLA < 3.5–4 mm2; MLA < 3.0 mm2 or 3–4 mm2 and plaque burden > 70%	Vessel dependent (MLA < 3.1 mm2 and area stenosis > 61% in proximal segments, MLA < 2.0 mm2 and area stenosis > 73%in non-proximal segments); MLA < 3.0 mm2		10,11,12
ACC/AHA/SCAI Guideline Recommendation	Class I: For procedural guidance to reduce ischemic events in ACS, particularly in LM or complex lesions (2025) Class IIa: For procedural guidance and to reduce ischemic events in patients undergoing coronary revascularization, particularly in LM or complex lesions (2021) Class IIa: To determine the mechanism of stent failure in patients with stent failure (2021) Class IIa: To help define lesion severity in patients with intermediate LM stenosis (2021)	Class I: For procedural guidance to reduce ischemic events in ACS, particularly in LM or complex lesions (2025) Class IIa: Reasonable alternative to IVUS for procedural guidance and to reduce ischemic events in patients undergoing coronary revascularization, except in ostial LM disease (2021) Class IIa: To determine the mechanism of stent failure in patients with stent failure (2021)		44,45
ESC Guideline Recommendation	Class I: When performing PCI on anatomically complex lesions in patients with chronic coronary artery disease, in particular LM stem, true bifurcations, and long lesions (2024) Class IIa: To evaluate the severity of intermediate stenoses of LM stem prior to revascularization (2024) Class IIa: To guide PCI in patients with ACS (2023) Class IIb: In patients with ACS and ambiguous culprit lesions (2023)	Class I: When performing PCI on anatomically complex lesions in patients with chronic coronary artery disease, in particular LM stem, true bifurcations, and long lesions (2024) Class IIa: To guide PCI in patients with ACS (2023) Class IIb: In patients with ACS and ambiguous culprit lesions, preferred modality (2023)		7,46
ISHLT Guideline Recommendation	Class IIa: Baseline IVUS in conjunction with coronary angiography at 4 to 6 weeks and one year after heart transplant to exclude donor transmitted or derived CAD, to detect rapidly progressive CAV, and provide prognostic information	Class IIa: Baseline OCT in conjunction with coronary angiography at 4 to 6 weeks and one year after heart transplant to exclude donor transmitted or derived CAD, and provide prognostic information		47

*IVUS*, Intravascular ultrasound; *OCT*, optical coherence tomography; *NIRS*, Near-infrared spectroscopy; *N/A*, not applicable; *PCI*, Percutaneous Coronary Intervention; *MSLA*, minimal stent luminal area; *MRLA*, minimal reference luminal area; *LM*, left main; *LAD*, left anterior descending coronary artery; *MLA*, minimal luminal area; *ACS*, acute coronary syndrome; *ACC*, American College of Cardiology; *AHA*, American Heart Association; *SCAI*, Society for Cardiovascular Angiography and Interventions; *ESC*, European Society of Cardiology; *ISHLT*, International Society for Heart and Lung Transplantation; *CAV*, Cardiac allograft vasculopathy

## Clinical Applications and Key Trials

### Definition of Ambiguous Angiographic Findings and Assessment of Intermediate Lesions

In patients with acute coronary syndrome (ACS) and no obvious angiographic culprit lesion, intravascular imaging can define potential coronary atherothrombotic causes, such as plaque rupture, non-obstructive erosions, calcified nodules, or napkin-ring lesions [9]. These diagnoses carry implications for therapy as continuation of antithrombotic and antiplatelet agents may be desirable in these settings. There are no randomized studies to determine the benefit of PCI in non-critical obstructive disease, thus the justification to proceed with revascularization must be made on clinical grounds, considering procedural risks related to lesion location (distal or bifurcation disease) and calcification, as well as vessel tortuosity and diameter. In patients already undergoing PCI, intravascular imaging may be useful to characterize intraprocedural ambiguous angiographic findings, such as haziness or luminal narrowing post-stent deployment or at the edges of the stent, which usually may be signs of unrecognized edge or vessel dissection, residual thrombus, or stent under-expansion.

Intracoronary imaging may be necessary to diagnose spontaneous coronary artery dissection (SCAD) when there is angiographic uncertainty [10]. However, it should be carefully considered given the high risk of propagating the dissection due to instrumentation into the false lumen. While there are no randomized studies that guide management of SCAD, most observational studies and expert consensus documents support a conservative approach [11]. If SCAD is strongly suspected by angiography and conservative therapy has been chosen, there is no indication to perform further intravascular imaging.

Some studies collectively highlight the role of IVUS and OCT in assessing intermediate coronary stenoses (40–70% occlusion by visual estimation on coronary angiography) for aiding in the decision to proceed with PCI. Koo et al. [12] and Waksman et al. [13] determined that there was only a modest correlation between minimal-lumen area (MLA) and plaque burden by IVUS and fractional flow reserve (FFR) values. The MLA thresholds that correlated with ischemia varied by location of the lesion. In both studies, an MLA ≤ 3.0 mm^2^ in the LAD and ≤ 2.4 mm^2^ in non-LAD arteries correlated with ischemia by FFR. Vergallo et al. [14] performed a multicenter, international, pooled analysis of individual patient-level data from published studies assessing FFR and OCT on the same vessel. In their study, an MLA < 2.0 mm^2^ and an area stenosis > 73% were effective predictors of an FFR ≤ 0.80. For proximal coronary segments, the optimal OCT thresholds that correlated with an FFR ≤ 0.80 were MLA < 3.1 mm2 and area stenosis > 61%. Although these studies point in general to some correlation between imaging findings and a threshold FFR, their findings were not perfectly aligned, suggesting that IVUS and OCT alone may not fully replace FFR for physiologic assessment and that if used as surrogates for this purpose, ample consideration should be made to the clinical context.

### PCI Optimization

The benefit of intravascular imaging to support and optimize PCI results and improve patient outcomes is now well established, yet use of these modalities remains low. Fazel et al. [15] recently reported that among Medicare beneficiaries undergoing PCI, use of intravascular imaging increased over a span of 6 years from 9.4% to 15.4%. In this population, use of intravascular imaging to guide PCI was associated with lower rates of major adverse cardiovascular events (MACE) (26% risk reduction), including all-cause mortality, compared to angiography-guided PCI. Similarly, Malik et al. [16] reported that the median use of intravascular imaging to guide PCI in the US had increased from 2.7% in 2016 to just 6.3% in 2020, despite imaging being available in 86% of hospitals. Although studies like these consistently show an increase in utilization of imaging, adoption continues to lag behind a growing body of evidence.

The IVUS-XPL Trial [17, 18] randomized patients to IVUS-guided versus angiography-guided drug-eluting stent implantation in long coronary lesions. IVUS guidance significantly reduced the rate of MACE, mainly by lowering target lesion revascularization (TLR)(2.5% vs 5.0% in the angiography-guided group, HR, 0.51 [95% CI, 0.28 to 0.91], *P* = 0.02). This benefit was sustained at 5 years with lower rates of MACE (5.6% vs 10.7%, HR: 0.50; 95% confidence interval: 0.34 to 0.75; *p* = 0.001) and TLR in the IVUS group (4.8% vs 8.4%, HR: 0.54; 95% CI: 0.33 to 0.89; *p* = 0.007). The ULTIMATE Trial [19, 20] was a large scale, all-comers trial comparing IVUS-guided and angiography-guided PCI using drug-eluting stents. IVUS guidance resulted in a significantly lower rate of target vessel failure (TVF) at 3 years (6.6% vs 10.7%, *p* = 0.01), primarily due to reduced Target Vessel Revascularization (TVR) (4.5% vs 6.9%, *p* = 0.05) and definite/probable stent thrombosis (0.1% vs 1.1%, *p* = 0.02). These studies consistently demonstrate that IVUS-guided PCI improves long-term clinical outcomes compared to angiography guidance alone.

The CLI-OPCI II multicenter registry [21] and a subsequent review of the major studies evaluating the clinical impact of OCT findings during PCI [22], revealed that in-stent MLA < 4.5 mm^2^, major distal edge dissections (> 200 μm in depth or > 60° and/or > 3 mm in length), and reference lumen narrowing < 4.5 mm^2^, were independent predictors of MACE. Suboptimal stent implantation utilizing these and other criteria occurred in 31%−58% of cases. Their findings underscored the value of OCT in detecting suboptimal stent deployment, which is linked to adverse clinical outcomes during follow-up. The DOCTORS Study [23] assessed the impact of OCT guidance in patients with non-ST-elevation acute coronary syndrome (NSTE-ACS) and reported that OCT-guided PCI led to larger final minimal stent area (MSA) and greater post-PCI FFR compared to angiography-guided PCI. ILUMIEN III [24] compared OCT-guided, IVUS-guided, and angiography-guided PCI and found that OCT guidance resulted in a similar MSA compared to IVUS and was superior to angiography in detecting suboptimal stent implantation. However, there was no significant difference in clinical outcomes between the imaging modalities at one year [25]. Its follow-up study, ILUMIEN IV [26] compared OCT-guided vs. angiography-guided PCI in patients receiving drug-eluting stents. Although OCT guidance led to larger stent dimensions, reduced residual stenosis, and significantly lower incidence of definite/probable stent thrombosis vs angiography guidance (0.5% vs. 1.4%; HR 0.36; 95% CI, 0.14–0.91; *P* = 0.02), there was no significant difference in the rate of the primary efficacy endpoint of target-vessel failure (death from cardiac causes, target-vessel myocardial infarction (MI), or ischemia-driven TVR). In the OCTOBER trial [27], OCT-guided PCI in patients with complex coronary bifurcation lesions was associated with a lower risk of a composite of death from a cardiac cause, target-lesion MI, or ischemia-driven TLR at 2 years than angiography-guided PCI (10.1% vs. 14.1%; HR 0.70; 95% CI, 0.50–0.98; *P* = 0.035). The collective findings of all these studies suggest that OCT guidance during PCI can generally improve procedural results and in certain patient populations improve long term outcomes compared to angiography guidance alone.

A few studies have performed head-to-head comparisons of IVUS and OCT for PCI guidance. In the OPINION Trial [28], use of both imaging techniques led to similar 1-year clinical outcomes with very low rates of 8-month angiographic binary in-segment restenosis (6.2% and 6.0%) and 12-month TVF (5.2% and 4.9%) for OCT and IVUS, respectively. Similarly, the OCTIVUS trial [29] reported that OCT-guided PCI was noninferior to IVUS-guided PCI with respect to the incidence of a composite of death from cardiac causes, target vessel–related myocardial infarction, or ischemia-driven TVR at 1 year. The RENOVATE-COMPLEX-PCI trial [30] demonstrated that either IVUS or OCT-guided PCI significantly reduced MACE compared to angiography-guided PCI in patients with complex coronary artery lesions. Over a 2.1-year follow-up**,** the imaging-guided group had a lower incidence of cardiac death, target-vessel myocardial infarction, and target-vessel revascularization (7.7% vs. 12.3%; HR 0.64; *P* = 0.008). Several recent meta-analyses and observational studies [31–34] have reported that both IVUS and OCT outperform angiography alone in PCI guidance by reducing stent-related complications and improving procedural outcomes. Although OCT may be superior for procedural optimization (stent expansion, malapposition detection), the current evidence for reducing long-term adverse events (stent thrombosis, restenosis) may be stronger for IVUS. There are no studies utilizing NIRS for optimization of PCI. Regardless of the individual choice in imaging, the preponderance of evidence supports using adjunct intravascular imaging with either IVUS or OCT over angiography-guidance alone as a superior strategy for optimizing stent implantation and improving long-term PCI outcomes, mainly lower rates of restenosis, target lesion failure (TLF), and stent thrombosis.

### Detection and Treatment of Vulnerable Plaque

Detection of vulnerable plaque to prevent future ACS and sudden death remains the holy grail of intravascular imaging. All three commercially available imaging technologies—IVUS, OCT, and NIRS, can detect at-risk plaques which are defined differently and correlated with different histopathologic findings. The PROSPECT trial [5] reported that the IVUS findings of plaque burden ≥ 70%, a luminal area ≤ 4mm^2^, and the presence of a virtual histology thin-cap-fibroatheroma (VH-TCFA) in non-culprit lesions were independent predictors of MACE (HR 5.03, 95% CI 2.51–10.11,*P* < 0.001, for plaque burden; HR 3.21, 95% CI 1.61–6.42, *P* = 0.001, for MLA; and HR 3.35, 95% CI 1.77–6.36, *P* < 0.001 for VH-TCFA). The presence of all three findings in a non-culprit lesion was associated with a 18.2% 3-year MACE rate compared to 1.9% without these characteristics (lesion HR 11.05, CI 4.39–27.82, *p* < 0.001). However, the main driver of MACE was rehospitalization for angina and not necessarily MI or death. ​ ​The LRP study [35] investigated the use of NIRS-IVUS to identify patients and plaques vulnerable to future coronary events. In this prospective cohort study, imaging of non-culprit segments was performed in patients undergoing cardiac catheterization for possible percutaneous coronary intervention. Over a two-year follow-up, the cumulative incidence of non-culprit MACE was 9%. The study found that for each 100-unit increase in the maximum lipid core burden index over a 4 mm segment (maxLCBI₄ₘₘ), there was an 18% increase in the risk of non-culprit MACE at the patient level, and a 45% increase at the plaque level. Patients with a maxLCBI₄ₘₘ greater than 400 had a significantly higher risk of non-culprit MACE compared to those with lower values (adjusted HR 3·39, CI 1·85–6·20, *p* < 0·0001). PROSPECT II [36] also used combination NIRS-IVUS to identify non-obstructive coronary plaques at risk of causing future adverse cardiac events. Over a median follow-up of 3.7 years, lesions with high lipid content detected by NIRS and large plaque burden identified by IVUS were found to be independent predictors of MACE. Specifically, lesions exhibiting both characteristics had a 4-year MACE rate of 7.0% (95% CI 4·0–10·0), and patients with at least one such lesion had a 13.2% MACE rate (95% CI 9·4–17·6). These findings suggest that combined NIRS-IVUS imaging can effectively detect vulnerable plaques and patients at increased risk for future cardiac events.

The CLIMA study [37] investigated the relationship between specific plaque characteristics in the proximal left anterior descending artery, as identified by OCT, and the occurrence of MACE over a 12-month period. The study found that the independent or simultaneous presence of four high-risk OCT features—MLA < 3.5 mm^2^, fibrous cap thickness (FCT) < 75 µm, lipid arc circumferential extension > 180°, and OCT-detected macrophages—was significantly associated with an increased risk of MACE. Patients exhibiting all four features had a hazard ratio of 7.54 (95% CI 3.1–18.6) for experiencing events such as cardiac death or target segment myocardial infarction, underscoring the prognostic value of OCT in detecting vulnerable plaques and stratifying patient risk.

The findings from these studies collectively suggest that NIRS, IVUS, and OCT imaging is a safe and effective tool for identifying vulnerable patients and plaques. The next question is whether identification of these lesions can be used to potentially guide preventive strategies in clinical practice. In a sub study of the PROSPECT Trial, Stone et al. [38] explored the concept of PCI for treating vulnerable coronary atherosclerotic non-obstructive plaques identified by IVUS-NIRS with a bioabsorbable scaffold (BVS). Their findings demonstrated that lesions treated with BVS had a statistically significantly larger follow-up MLA, their primary endpoint, compared to those managed with guideline directed medical therapy alone. Although TLF rates at 24 months were similar between the groups, the study was not powered to detect differences in clinical events. Their results suggested that PCI of angiographically mild lesions deemed to be vulnerable is safe, and highlighted the need for larger trials to assess the clinical efficacy of preemptive PCI in vulnerable plaques.​ The PREVENT trial [39] evaluated whether preventive PCI of non-flow-limiting vulnerable plaques, identified via intracoronary imaging, could improve clinical outcomes compared to optimal medical therapy (OMT) alone. At the 2-year mark, the primary composite endpoint of cardiac death, target-vessel myocardial infarction, ischemia-driven TVR, or hospitalization for unstable or progressive angina, occurred in 0.4% of the PCI group versus 3.4% of the OMT group (absolute difference: −3.0 percentage points; *p* = 0.0003). These findings suggest that in patients with non-flow-limiting vulnerable plaques, preventive PCI in addition to OMT may reduce major adverse cardiac events compared to OMT alone. Important to note that in this study, operators employed IVUS, OCT, or NIRS based on their expertise and discretion to identify vulnerable lesions. A plaque was classified as vulnerable if it met at least two of the following criteria: MLA ≤ 4.0 mm^2^ by IVUS or OCT, plaque burden > 70% by IVUS, maxLCBI₄ₘ > 315 identified through NIRS, and the presence of a TCFA detected by OCT or VH-IVUS. This was the first large trial to show the potential effect of the focal treatment for vulnerable plaques identified by intravascular imaging and gives further support to the paradigm of prevention through early detection and treatment of non-flow-limiting, high-risk vulnerable plaques.

### Intravascular Imaging in Special Populations

In heart transplant recipients, IVUS remains the gold standard for detecting diffuse intimal thickening and cardiac allograft vasculopathy (CAV) [40]. There are robust data that IVUS-based findings of intimal thickening are associated with clinical outcomes in heart transplant patients and have important prognostic implications. Besides several studies demonstrating an association between high-grade acute cellular rejection and abnormal OCT findings, hard outcome data validating the prognostic impact of OCT plaque parameters in the heart transplant population continue to emerge [41]. A recent study has utilized OCT for this purpose in a pediatric heart transplant population with promising results [42]. The totality of data have led to an updated recommendation in 2023 for use of OCT as an alternative to IVUS to detect CAD.

IVUS guidance during PCI of chronic total occlusions has been associated with higher procedural success and improved long-term outcomes compared with angiography alone. In a study by Tian et al. [43], in-stent late-luminal loss in the IVUS-guided group was significantly lower compared to the angiography-guided group at one-year follow-up (0.28 ± 0.48 mm vs. 0.46 ± 0.68 mm, *p* = 0.025), with a significant difference in restenosis of the “in-true-lumen” stent between the two groups (3.9% vs.13.7%, *p* = 0.021). Kim et al. [44] reported that the major MACE rates (composite of cardiac death, MI, and TVR) were significantly lower in the IVUS-guided group than that in the angiography-guided group (2.6% versus 7.1%; *P* = 0.035; HR 0.35; 95% confidence interval, 0.13–0.97). However, the rate of cardiac death or TVR was not significantly different between the groups.

## Guideline Recommendations for Intravascular Imaging

The 2021 American College of Cardiology/American Heart Association/Society of Cardiac Angiography and Interventions (ACC/AHA/SCAI) Guidelines for Coronary Artery Revascularization [45] recommend that in patients undergoing coronary stenting, IVUS can be useful for procedural guidance and to reduce ischemic events, particularly in cases of left main or complex coronary artery stenting (Class IIa recommendation). OCT is a reasonable alternative to IVUS for procedural guidance, except in ostial left main disease (Class IIa recommendation). In patients with stent failure, IVUS or OCT is reasonable to determine the mechanism of stent failure (Class IIa recommendation). The newer 2025 ACC/AHA/SCAI Guidelines for management of patients with acute coronary syndromes [46] upgraded the use of intracoronary imaging with IVUS or OCT for procedural guidance to a Class I recommendation to reduce ischemic events, particularly in left main or complex lesions.

The 2024 European Society of Cardiology (ESC) Guidelines [47] recommend use of intracoronary imaging guidance by IVUS or OCT when performing PCI on anatomically complex lesions in patients with chronic coronary syndromes, in particular left main stem, true bifurcations, and long lesions (Class I recommendation). The 2023 ESC Guidelines for the management of ACS [9] still recommend that intravascular imaging should be considered to guide PCI as a Class IIa recommendation, however it seems likely this guideline will be updated to match its American counterpart. The guidelines further note that intravascular imaging (preferably OCT) may be considered in patients with ambiguous coronary lesions (Class IIb recommendation). Currently, there is no recommendation for the use of NIRS imaging in the ACC, AHA or ESC guidelines.

The 2023 International Society for Heart and Lung Transplantation (ISHLT) Guidelines [48] endorse consideration of baseline IVUS imaging in conjunction with coronary angiography at 4 to 6 weeks and one year after heart transplant to exclude donor transmitted or derived CAD, to detect rapidly progressive CAV, and provide prognostic information as a Class IIa recommendation in adults and Class IIb in pediatric recipients. In the adult population, OCT in conjunction with coronary angiography at 4 to 6 weeks and one year after heart transplant may be considered to exclude donor transmitted or derived CAD and provide prognostic information (Class IIa recommendation).

## Future Directions

### Artificial Intelligence (AI) and Machine Learning

The integration of AI and machine learning in intracoronary imaging has been utilized to some degree for automating plaque detection and image interpretation and characterizing lesion morphology. Newer systems that offer automated interpretation and morphometry are already commercially available [49]. Advances in deep learning-based segmentation models and AI-assisted image interpretation of OCT and IVUS have demonstrated high accuracy in plaque characterization, image segmentation for lumen area and plaque burden, and stent assessment [50]. AI-driven algorithms will likely reduce operator variability, improve risk stratification and predictive models, and streamline clinical decision-making by incorporating clinical and imaging information that will lead to more personalized and specific treatment strategies [51].

### Next-Generation Imaging Systems

Further advancements in high-definition IVUS and next-generation OCT are expected to improve image resolution, penetration depth, and signal clarity, allowing for even more precise assessments of coronary lesions. Co-registration of the angiogram with information derived from intravascular imaging and automated interpretation can now create comprehensive coronary roadmaps that are readily available and continue to improve. Multimodality systems that provide higher resolution and enhanced depth penetration of OCT and NIRS co-registration can avoid the need to use contrast displacement of blood and may improve plaque characterization and PCI-guidance [52]. Similarly, other emerging hybrid intravascular devices can overcome limitations of standalone imaging modalities and may be suited for special applications. OCT-near-infrared fluorescence imaging is an evolving technique that enables real-time visualization of inflammation in atherosclerotic plaques, which may further enhance risk assessment of plaques deemed vulnerable by detecting macrophage-rich, highly inflamed lesions [53]. Furthermore, integration of computational fluid dynamics (CFD) models with imaging data could enhance functional assessments, bridging the gap between anatomic and physiologic lesion evaluation [54].

### Preventive PCI Trials

Despite growing evidence that high-risk plaques identified by intravascular imaging contribute to future adverse events, the role of pre-emptive PCI in treating these non-flow-limiting lesions remains under investigation. More large-scale, randomized trials are under way to confirm the hypothesis that targeting high-risk plaques with advanced imaging-guided PCI can prevent myocardial infarction and ACS-related sudden death [55–57]. Beyond PCI, strategies of intensification of systemic pharmacotherapy or targeted local delivery could be designed and tested. Future research will focus on refining patient selection criteria, plaque-specific risk-stratification, plaque-tailored therapy, optimal stent deployment strategies, and adjunctive pharmacological therapies to enhance preventive interventions.

## Conclusions

Intravascular imaging has emerged as a transformative tool in the diagnosis and management of coronary artery disease. By overcoming the limitations of conventional angiography, modalities such as IVUS, OCT, and NIRS enable precise lesion characterization, guide optimal PCI, and facilitate patient and plaque-specific risk stratification. Landmark trials continue to demonstrate that imaging-guided strategies lead to superior procedural outcomes and offer critical prognostic insights, validating the use of intravascular imaging and of the imaging paradigm. The evidence supporting adjunct use of intravascular imaging to optimize PCI results and improve outcomes continues to accumulate to the point that some are starting to advocate use of imaging as a performance measure for PCI [58]. As technology keeps evolving at a rapid pace, further integration of intracoronary imaging modalities with AI for ease of interpretation and enhanced prediction is poised to open new avenues for preventive interventions and improved long-term outcomes in CAD.

## Key References

- Lee JM, Choi KH, Song YB, et al. RENOVATE-COMPLEX-PCI Investigators. Intravascular imaging-guided or angiography-guided complex PCI. N Engl J Med 2023;388:1668–1679.
  - **This study demonstrated that either IVUS or OCT-guided PCI significantly reduced MACE compared to angiography-guided PCI in patients with complex coronary artery lesions**.
- Park SJ, Ahn JM, Kang DY, et al. Preventive percutaneous coronary intervention versus optimal medical therapy alone for the treatment of vulnerable atherosclerotic coronary plaques (PREVENT): a multicentre, open-label, randomised controlled trial. The Lancet 2024;403:1753–1765.
  - **This is the first large trial to show the potential effect of the focal treatment for vulnerable non-flow limiting plaques identified by intravascular imaging**.

## Data Availability

No datasets were generated or analysed during the current study.
